# Development of a mobile 3D printer and comparative evaluation against traditional gantry systems

**DOI:** 10.1007/s10845-024-02433-z

**Published:** 2024-06-06

**Authors:** Abdullah Alhijaily, Abdulrahman Alqarni, Zekai Murat Kilic, Paulo Bartolo

**Affiliations:** 1 Department of Mechanical Engineering, Taibah University, Taibah, Saudi Arabia; 2 Department of Mechanical Engineering, King Khalid University, Abha, Saudi Arabia; 3 Department of Mechanical, Aerospace and Civil Engineering, The University of Manchester, Manchester, UK; 4 Singapore Centre for 3D Printing, School of Mechanical and Aerospace Engineering, Nanyang Technological University, Nanyang, Singapore

**Keywords:** 3D printing, Mobile 3D printer, Mobile robot, Accurate mobile robot, Mobile 3D printing

## Abstract

Fixed robots have dominated the market of additive manufacturing (AM), despite presenting several limitations, such as the stationary nature of these robots and the limited workspace. Mobile robots solve these problems as they can move freely in the printing area without being rooted to the ground. This allows mobile robots to print large-scale structures and print in places that are unsafe for humans to reach and deploy fixed robots. However, mobile robots suffer from poor positional accuracy. In this paper, we present an accurate mobile robot for material extrusion AM and discuss in detail the design of the mobile 3D printer and its components. This work is the first to rigorously compare the quality, accuracy, and mechanical properties of parts printed by the mobile 3D printer against those printed by gantry systems. Results show that the parts produced by the proposed system are comparable to those of a gantry system in certain aspects such as the overall quality and shape fidelity. Additionally, the accuracy exceeded the state-of-the-art of mobile 3D printing achieving low ranges of less than 0.5 mm. Moreover, the proposed system outperforms other plastic 3D printing mobile robots in literature, excelling in both quality and accuracy.

## Introduction

Mobile robots have been extensively used in manufacturing (Oztemel & Gursev, 2018), being particularly relevant for flexible manufacturing systems consisting of multiple machines, material handling devices, and storage units (Dang et al., 2018). In such systems, mobile robots are being used to perform material handling and transportation tasks without the need to alter the manufacturing environment (Datta et al., 2007). Beyond mere facilitation, mobile robots actively participate in the manufacturing process itself. Instead of constructing large, custom machine tools for extensive manufacturing operations, compact tools have been innovatively designed for mobile robots to move around the part (Shneier & Bostelman, 2015). This innovative approach optimizes resource allocation without compromising the quality or scale of production. Moreover, mobile robots have ventured into performing an array of manufacturing processes, including polishing, drilling, grinding, and milling (Tao et al., 2019), further establishing their versatility and indispensability in modern manufacturing systems.

Recently, the use of mobile robots for Additive Manufacturing (AM) attracted significant academic and industrial attention due to several advantages they offer such as portability, large-scale fabrication, and printing on locations unreachable by humans (Troemner et al., 2022). These benefits are unobtainable using traditional additive manufacturing systems such as gantry robots and robotic arms (Alhijaily et al., 2024). For example, the workspace of a gantry robot is limited to a portion of the size of the robot itself. In addition, fixed robots are disadvantageous in logistics compared to mobile robots. Therefore, several publications reported the use of mobile robots in AM for a wide range of domains such as in concrete printing (Zhang et al., 2018), material handling (Arrais et al., 2019), and structure reinforcement (Hack et al., 2020). However, mobile 3D printing faces several challenges, such as maintaining high motion accuracy, ensuring machine independence for axis movement, and addressing issues of structural rigidity. Solving the poor accuracy of mobile robots is particularly important for manufacturing processes such as AM.

Currently, there are three main approaches for mobile robots in AM: (i) move-print, (ii) print-move-print, and (iii) print-while-moving approaches. In the move-print approach, mobile robots are only used for the initial transportation of printing equipment. In this approach, a mobile robot transports the 3D printer, often a robotic arm, directly to the designated site, where printing proceeds. An example of this approach is the Digital Construction Platform (DCP), developed by Keating et al. (2017). This platform stands as a fully automated mobile robotic system with the capabilities to carry out construction tasks onsite. Equipped with a robotic arm specifically designed for concrete 3D printing, the DCP is capable of printing large and dome-like structures. Similarly, Zhang et al. (2018) implemented this concept in cooperative printing of a large-scale concrete structure. In this case, authors deployed two holonomic mobile robots, each equipped with a 6-axis robotic arm for the printing operation. The mobile robots localize their pose in the environment and move to a designated printing location at which the robotic arm takes full control and starts the printing process. The move-print approach is the simplest approach for using mobile robots in AM, as the mobile robot is only controlled once, but such kind of systems do not fully utilize the mobility aspect of mobile robots.

Another approach for using mobile robots with 3D printing is the print-move-print approach. In this approach, the mobile robot moves between the printing process to change location and to increase the reach of the printing system. Li et al. (2021) developed a system called Simultaneous Localization and Additive Manufacturing based on the use of a mobile printing system that prints a specific region of a part at a stationary position, then moving to another position to print another region, repeating this sequence until the part is printed. The localization is determined using a 3D scanner alongside a total station. Similar works were conducted by Lachmayer et al. (2022) that developed a mobile manipulator that moves between prints for concrete 3D printing, and the *in-situ* fabricator developed by Thoma et al. (2017). However, this print-move-print approach presents several limitations. One limitation is that the printed part must be segmented as the printing process is not continuous, being interrupted by the movement of the mobile robot. Moreover, the frequent moves, stops, and prints significantly increase the overall printing time.

A more sophisticated approach is the print-while-moving in which the mobile robot actively moves during the printing process. This approach was explored by Tiryaki et al. (2020) using mobile manipulators for concrete 3D printing to print large structures, larger than the reach of the robotic arm mounted on the mobile platform. Other relevant examples are the work of Sustarevas et al. (2019, 2018, 2022) that developed multiple print-while-moving mobile manipulator systems, including the YouWasps platform for cooperative concrete 3D printing. The print-while-moving approach is more common in plastic 3D printing, due to the small size of the mobile robots, leading to the use of Z-axis stages rather than heavy and large multi-axis robotic arms, forcing the mobile robot to perform the planar motion (Alhijaily et al., 2023; Duan et al., 2019; Marques et al., 2017; Milkert, 2014). Marques et al. (Marques et al., 2017) in their work on cooperative printing for plastics developed a mobile robot with a fixed Z-axis stage. Motion in the X-Y plane was performed by the holonomic mobile robot while the Z-axis was controlled by the mounted Z stage. Similarly, Xu et al. (2021) implemented a print-while-moving strategy using a nonholonomic mobile 3D printer with a custom-mounted arm.

However, mobile robots in AM, based on material extrusion strategies, are limited in terms of positioning accuracy due to numerous contributing factors, such as slipping and skidding (Borenstein et al., 1997). This poses some limitations regarding the use of mobile robots for high-accuracy 3D printing applications. However, accuracy requirements differ based on the size of the nozzle. Concrete 3D printing is more tolerant as typical nozzle sizes are in the centimeters range (Khan, 2020). For example, Tiryaki et al. (2020) reported a maximum positional discrepancy of 9.90 mm in their concrete mobile 3D printing setup. Similarly, in filament-based extrusion AM, Marques et al. (2017) identified an average positional deviation of 1.23 mm, which is significant given that the diameter of the filament is 1.75–2.85 mm (Gancheva et al., 2021), and typical filament-based extrusion gantry machines exhibit a micron range accuracy (Melenka et al., 2015). This issue is evident in the quality of the parts produced by plastic mobile 3D printers, as seen in parts fabricated by (Marques et al., 2017; Milkert, 2014; Xu et al., 2021).

As observed, the adoption of mobile robots for AM has been hampered by significant challenges, notably the poor accuracy of mobile robots and the lack of comparative studies with traditional gantry systems. This research seeks to bridge these gaps by proposing a novel, accurate mobile robot for filament-based material extrusion that utilizes a print-while-moving approach. This mobile robot is shown to significantly improve both quality and accuracy of 3D printing in mobile platforms. Moreover, this work presents several contributions, mainly:

- The developed mobile robot significantly enhances positional accuracy in mobile 3D printing, producing good-quality parts outperforming other plastic mobile 3D printers.
- The use of the HTC Vive system for accuracy demanding applications such as AM, which was not fully explored before for such purposes.
- This is the first work that presents a comprehensive comparison between mobile robots and gantry systems, covering part quality, dimensional accuracy, mechanical strength, and surface properties. Our findings show that the mobile robots produce parts of comparable quality to those printed by gantry systems.

These contributions underscore the significance of our work, addressing previously identified limitations in mobile 3D printing and setting a new direction for future research and development in the field.

## Mobile 3D printer

### System design

**Fig. 1:**
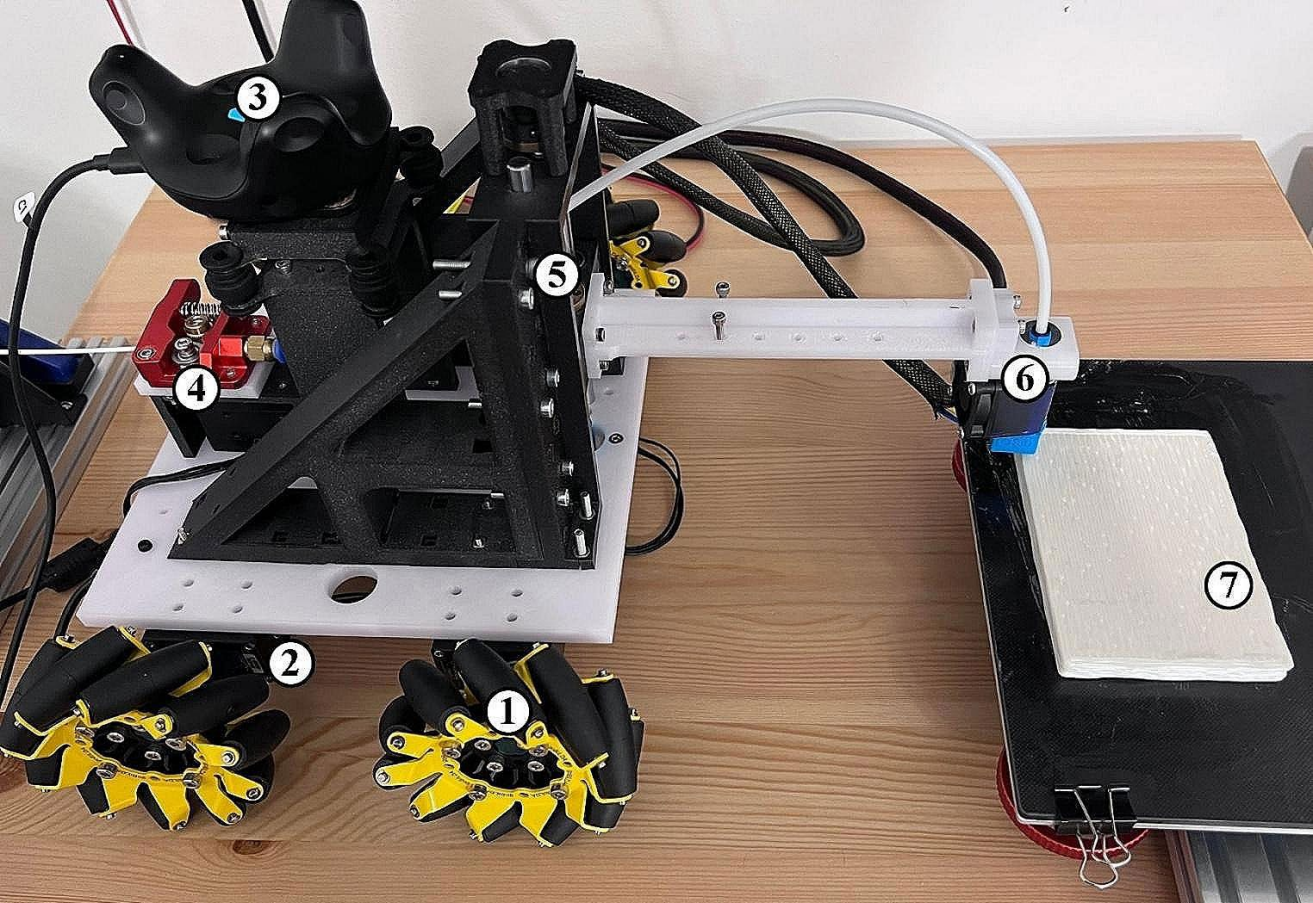
The developed mobile 3D printer. Legend: (1) mecanum wheels, (2) wheel motor, (3) Vive tracker, (4) E-axis, (5) Z-axis, (6) printhead, (7) printed part on the printing platform

The mobile robot system (Fig. 1) was designed for filament-based extrusion AM, and comprises motion, heating, and cooling components. The system incorporates four wheels (goBILDA, USA) that facilitate movement along the X-Y axis, driven by a single Dynamixel XH430-W350 DC motor (Dynamixel, South Korea). Vertical movement (Z-axis) of the printhead is achieved through an 8 mm threaded rod (Misumi, USA) connected to the motor, and supported by two 8 mm linear rods (Misumi, USA). To extrude the filament to the printhead (E-axis), an additional motor is utilized. The motors for both the Z and E axes mirror those powering the wheels, ensuring uniform control across the system and synchronized movements. Instead of only controlling the Z-axis motor during layer change, the position of the Z-axis is frequently modified based on the vertical vibration of the mobile robot. Due to noisy measurements and external factors (e.g. slipping and uneven terrain), the velocity of the robot slightly varies when performing the required motion. This requires the E-axis motor to be synchronized with the absolute velocity of the mobile robot. Extruding faster or slower than the current velocity of the mobile robot results in a non-uniform filament’s thickness. Thus, to ensure synchronized motion and uniform lines during the printing process, the controller that controls the velocity of the wheels also controls the E-axis motor based on the estimated velocity of the robot.

The printing system consists of cooling and heating elements. The cooling elements consist of two fans, one to cool the heatsink of the hotend and another that cools the printed filament, NF-A14 (Noctua, Austria). However, as fans are large sources of vibrations, the part cooling fan is positioned at a fixed place (out of the printhead) covering the entire printing area. A high nozzle size was used as large nozzles are less sensitive to accuracy fluctuations and noise. The nozzle is a CHT Bondtech Brass Nozzle (Bondtech, Sweden) with 1.8 mm nozzle diameter, the print line width was fixed at 1.4 mm and the layer height for all the prints was 1 mm. The hotend is E3D V6 (E3D, UK) which can reach temperatures of 300 °C and the heated bed is able to reach 120 °C. The heating and cooling elements are controlled using an MKS Gen 1.4 electronic board (Makerbase, China). The dimensions of the mobile platform are 220 mm by 220 mm, with a height of 180 mm. The mobile robot is tethered but can move freely in a circle with a 500 mm diameter.

Table 1 provides a detailed overview of the main hardware components used in the mobile robot and corresponding specifications.

**Table 1:** Overview of the hardware used in the mobile robot

Component	Specification
Wheels	96 mm diameter mecanum wheels with 10 rollers.
Wheel Motor	Dynamixel XH430-W350 DC motor
Z-Axis Structure	8 mm threaded rod and two 8 mm linear rods
Z-Axis Motor	Dynamixel XH430-W350 DC motor
E-Axis Motor	Dynamixel XH430-W350 DC motor
Fans	Heatsink fan and part cooling fan (NF-A14)
Hotend	E3D V6 with maximum temperature of 300 °C
Nozzle	1.8 mm Bondtech CHT Coated Brass Nozzle
Heated Bed	Creality heated bed with maximum temperature of 120 °C
Electronic Board	MKS Gen 1.4
Inertial Measurement Unit	LPMS-USBAL2 for linear acceleration and angular velocities measurements
Motor Encoder	AS5045 optical encoders
Optical Beacon	HTC Vive system with beacons and trackers

### Motion system

**Fig. 2:**
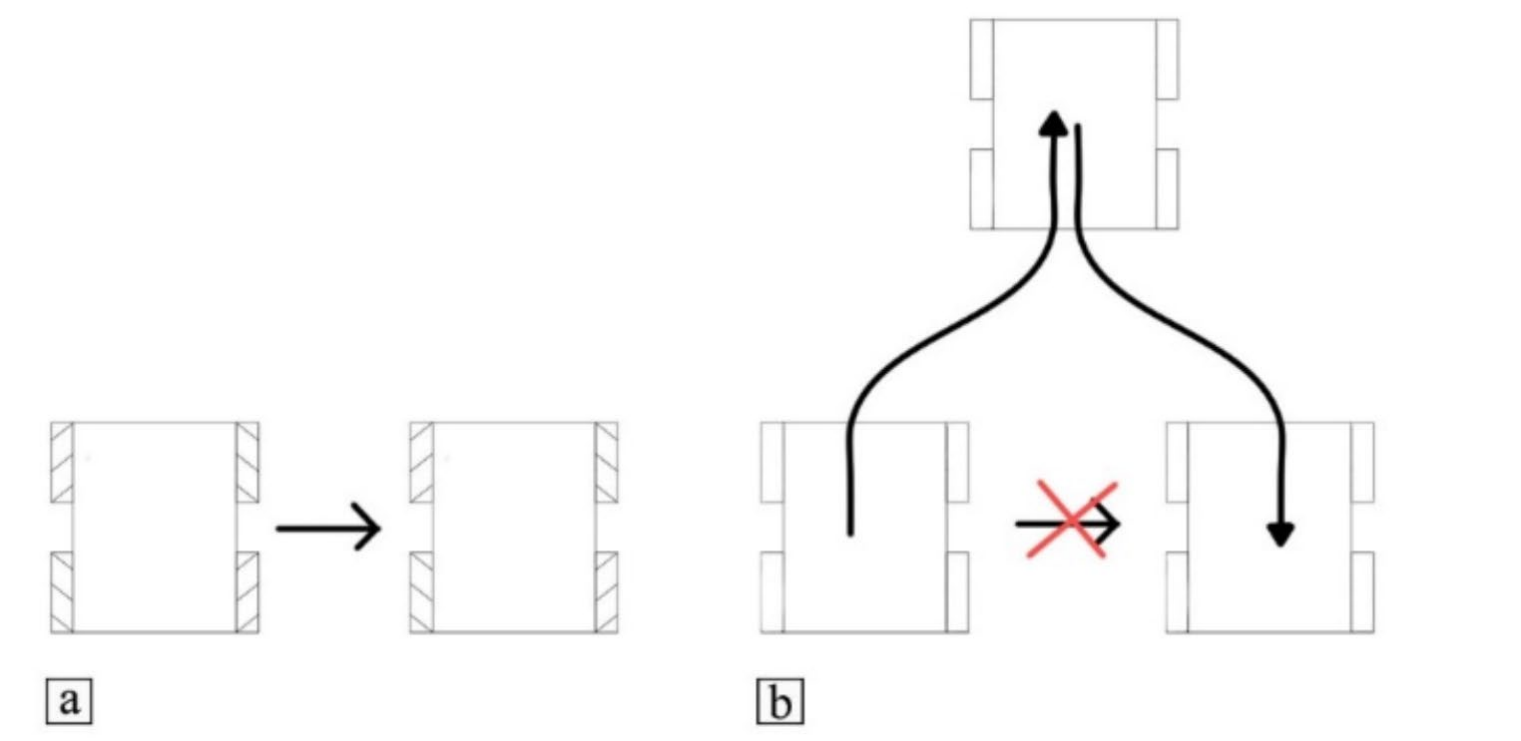
Moving sideways for mobile robots (**a**) Omnidirectional robots can move sideways (**b**) nonholonomic robots must take a different route to reach a similar position

In terms of velocity constraints on the body of the robot, mobile robots can be classified as holonomic and nonholonomic (Lynch & Park, 2017). Holonomic robots, also known as omnidirectional robots, do not have any constraints on the velocity. On the other hand, nonholonomic mobile robots are subjected to a Pfaffian constraint on the velocity (Lynch & Park, 2017), which prevents cars from moving sideways without rotating (Fig. 2). However, nonholonomic robots can reach any configuration in the configuration space, which means that this velocity constraint cannot be integrated into a position constraint. Thus, omnidirectional mobile robots are superior to nonholonomic ones for AM applications. Moreover, a mobile robot can be designed as an omnidirectional robot in many ways and one of them is by equipping the robot with omnidirectional wheels, which can be compared to equipping a robot with multiple legs and wheels (Sustarevas et al., 2018). Moreover, the implementation of omnidirectional wheels enables the design of more compact and simpler mobile robots. Therefore, the proposed mobile robot was designed as omnidirectional by using mecanum wheels.

**Fig. 3:**
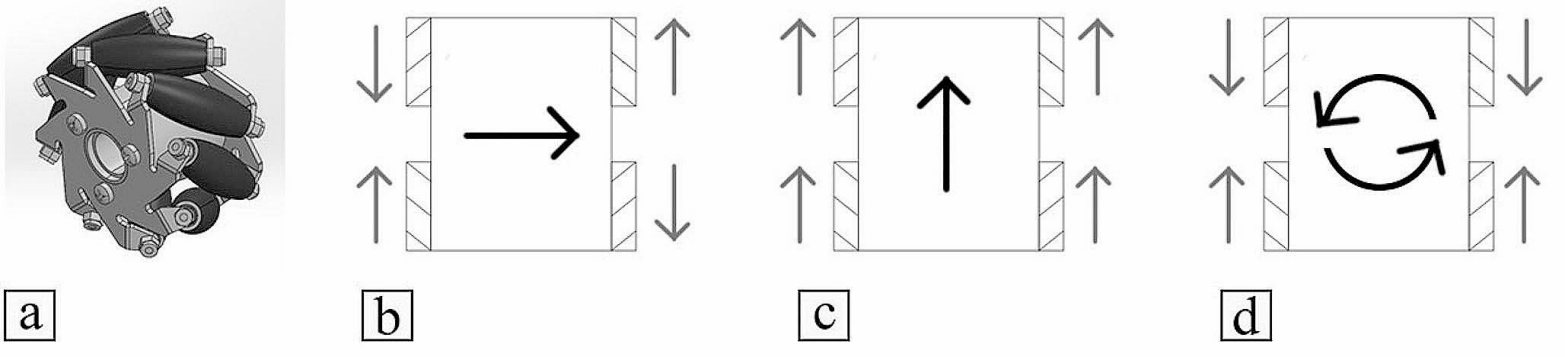
(**a**) A mecanum wheel. Wheels motions to move the robot in the (**b**) X-axis, (**c**) Y-axis (**d**) rotate around Z-axis

The core of a mecanum wheel (Fig. 3a) resembles that of a standard wheel, yet it is distinguished by the presence of small rollers mounted around its perimeter. The presence of the rollers allows the mobile robot to be omnidirectional. This can be achieved by independently controlling each wheel based on the required overall motion of the robot (Fig. 3b-d). These possibilities are not present in nonholonomic or car-like mobile robots.

In the case of mecanum wheels the kinematic equation that governs the rotation speed of each wheel to perform the required overall motion is given by the following equation (Lynch & Park, 2017):

$$\left[\begin{array}{c}{\dot{\theta }}_{w1}\\ {\dot{\theta }}_{w2}\\ {\dot{\theta }}_{w3}\\ {\dot{\theta }}_{w4}\end{array}\right]=\frac{1}{r} \left[\begin{array}{ccc}-1& 1& \left|{d}_{x}\right|+\left|{d}_{y}\right|\\ 1& 1& -\left|{d}_{x}\right|-|{d}_{y|}\\ 1& 1& \left|{d}_{x}\right|+\left|{d}_{y}\right|\\ -1& 1& -\left|{d}_{x}\right|-\left|{d}_{y}\right|\end{array}\right] \left[\begin{array}{c}{\dot{x}}_{p}\\ {\dot{y}}_{p}\\ \dot{\theta }\end{array}\right] =H{\dot{q}}_{p} \left(1\right)$$

where ${\dot{\theta }}_{wi}$ is the angular velocity of the $i$th wheel, $r$ is the radius of the wheel, ${d}_{x}$ and ${d}_{y}$ are the x and y distances, respectively, from the center of the platform to a wheel, and ${\dot{q}}_{p}=\left[{\dot{x}}_{p} {\dot{y}}_{p} {\dot{\theta }}_{p}\right]$ is the vector of linear and angular velocities of the mobile platform in the body frame.

The mecanum wheels used in the developed mobile robot are the 96 mm diameter goBILDA wheels with 10 rollers. However, this introduces some vertical vibrations as the mecanum wheels present a finite number of rollers in the circumference (normal wheels can be considered to have infinite rollers, i.e. no discontinuities in the wheel). The number of rollers around the circumference of the wheel affects both the frequency and magnitude of the vertical vibration. Fewer number of rollers increases the magnitude of the vibration, which in turn affects the accuracy of the mobile robot in the Z-axis, while a higher number of rollers reduces this effect. Nevertheless, as this effect is known beforehand it can be compensated by the control software, considering the effect of all the wheels combined.

### Localization and control

The localization system makes use of three different types of sensors to estimate the full pose of the mobile robot. The first sensor is the AS5045 optical encoder in each wheel (ams, Austria), which measures the corresponding rotational velocity. Furthermore, an Inertial Measurement Unit (IMU), LPMS-USBAL2 (LP-RESEARCH, Japan), is used to measure the linear acceleration and angular velocities of the mobile robot in the platform frame, supporting the communication system. Finally, global sensors are used since local sensors have several limitations when measuring the state of the robot, being affected for example by factors such as skidding, slipping, and backlash in the wheels. Moreover, both the encoders and the IMU accumulate errors over time.

Despite the fact that global sensors overcome many of the limitations of local sensors, there is a trade-off for using various global sensors. For example, Global Navigation Satellite Systems (GNSS) have low accuracy and do not work in indoor areas (Borenstein et al., 1997), Light Detection and Ranging (LiDAR) systems are costly and reach hundreds of millimeters in terms of accuracy (Triglav-Čekada et al., 2009) and accurate cameras are typically very expensive (Mautz & Tilch, 2011). The global sensor system used in the proposed mobile robot consists of two optical beacons with a receiver on the mobile robot. The main drawback of these sensors is the requirement of a line of sight between the emitter and the receiver. This drawback is avoided by placing the emitters on long rods and ensuring that the receiver on the robot is placed as high as possible.

The optical beacon system chosen was the HTC Vive (HTC, Taiwan), which is a system used for Virtual Reality (VR) applications. It comprises two important hardware elements: base stations and trackers (Wheeler et al., 2019). The base stations send infrared light across the room in sequence. The trackers capture this light signal using photodiodes and decode it to know the mobile robot’s relative position to the base stations. Trackers come in different shapes and forms, mainly head-mounted display (HMD), controllers, and Vive trackers that are mounted on different equipment. In our system, Vive trackers are used due to their lightweight, compact form factor, and ease of mounting on the mobile robot. Using two base stations allows the tracking area to be 5 m by 5 m while increasing the number to four base stations increases the tracking area to 10 m by 10 m. Moreover, the HTC Vive system showed high accuracy for localization (Borges et al., 2018). However, further testing is conducted in this paper, specifically focusing on the impact of the software employed and the chosen configuration settings.

The sensors used in the developed mobile robot complement each other, as there are downsides for each individual sensor. For example, the odometry data from the encoders are prone to external disturbances. The sensors are fused using Kalman Filter (KF) (Thrun et al., 2005), which is a probabilistic Gaussian filter used for state estimation of linear systems. The Kalman Filter (KF) functions on a two-step process - prediction and correction. The process begins by predicting the robot’s position using a motion model that incorporates data from the IMU. Subsequently, this prediction is refined through a measurement model that combines information from both the encoders and the Tracker, employing the kinematic model of Eq. (1). The motion and the measurement models are:

$$\begin{array}{c}{x}_{k}={A}_{k} {s}_{k-1}+{B}_{k} {u}_{k}+{w}_{k}\#\left(2\right)\end{array}$$

$$\begin{array}{c}{z}_{k}={C}_{k} {s}_{k}+{v}_{k}\#\left(3\right)\end{array}$$

Equation (2) is part of the prediction step in the KF process. It predicts the future state of the system based on the current state, the control input, and the process noise. On the other hand, Eq. (3) is part of the correction step, and it relates the predicted state to the actual measurements. In these equations ${s}_{k}$ is the vector containing the position and velocity of the robot, ${A}_{k}$ is the state transition matrix that specifies how the state changes over time, ${B}_{k}$ is the input matrix, ${u}_{k}$ is the control vector, ${z}_{k}$ is the measurement vector, ${C}_{k}$ is the measurement matrix, and finally the motion and measurement noises, ${w}_{k}$ and ${v}_{k}$, are found empirically by trial and error. In these equations subscript $k$indicates the variable at the current calculation step while $k-1$ is the previous step. The matrices ${A}_{k}$ and ${B}_{k}$ used in our implementation are given by:

$$\begin{array}{c}{\text{A}}_{\text{k}}= \left[\begin{array}{cccccc}1& 0& 0& {\Delta }{t}_{k}& 0& 0\\ 0& 1& 0& 0& {\Delta }{t}_{k}& 0\\ 0& 0& 1& 0& 0& {\Delta }{t}_{k}\\ 0& 0& 0& 1& 0& 0\\ 0& 0& 0& 0& 1& 0\\ 0& 0& 0& 0& 0& 1\end{array}\right] , \\{B}_{k}=\left[\begin{array}{ccc}0.5{\Delta }{{t}_{k}}^{2}& 0& 0\\ 0& 0.5{\Delta }{{t}_{k}}^{2}& 0\\ 0& 0& 0.5{\Delta }{{t}_{k}}^{2}\\ {\Delta }{t}_{k}& 0& 1\\ 0& {\Delta }{t}_{k}& 0\\ 0& 0& {\Delta }{t}_{k}\end{array}\right] \#\left(4\right)\end{array}$$

where ${t}_{k}$ is the current time step. The measurement matrix ${C}_{k}$ is given by:

$$\begin{array}{c} \\ {\text{C}}_{\text{k}}= \left[\begin{array}{cc}\text{I}& 0 \\ 0& H\end{array}\right] \#\left(5\right)\end{array}$$

${C}_{k}$ is a 7-by-7 matrix, H is the kinematic model shown in Eq. (1).

The KF algorithm (Thrun et al., 2005) takes as an input the previous state ${s}_{k-1}$ and its associated covariance ${P}_{k-1}$, along with the current control vector ${u}_{k}$ and the measurement vector ${z}_{k}$. The output of the algorithm is the estimated state ${s}_{k}$ with the updated covariance ${P}_{k}$

The control of the mobile robot is achieved through two control loops (Reis et al., 2022): the first is a trajectory-following controller operating at 500 Hz, which includes both the path planning and state estimation functions. The second, a low-level inner loop, runs at 8 kHz and controls the motors based on the motors’ encoders. The overall process goes as follows. The path planner sends the required trajectory to a module that generates a trapezoidal profile and converts the required body velocity to the velocities of the wheels. The internal controller then commands the motors to achieve the requested velocities. The loop to the outer controller is closed by measuring the state of the robot using the localization module, and then the errors are determined by comparing the state estimated to the requested trajectory of the path planner.

**Fig. 4:**
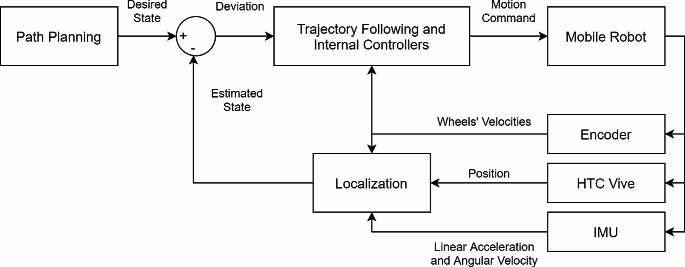
Control block diagram showing the feedback system

The control system, integrated with the localization module, forms a closed-loop feedback system, as shown in Fig. 4. This integration ensures real-time adjustments and precision in the mobile robot’s movement by continuously comparing the robot’s actual position with its intended path. The feedback system utilizes the sensor data to dynamically correct the robot’s trajectory, minimizing errors caused by slippage or uneven terrain. This allows for accurate and consistent material deposition, which is necessary for achieving 3D printed parts with good dimensional accuracies.

### Information flow and communication system

**Fig. 5:**
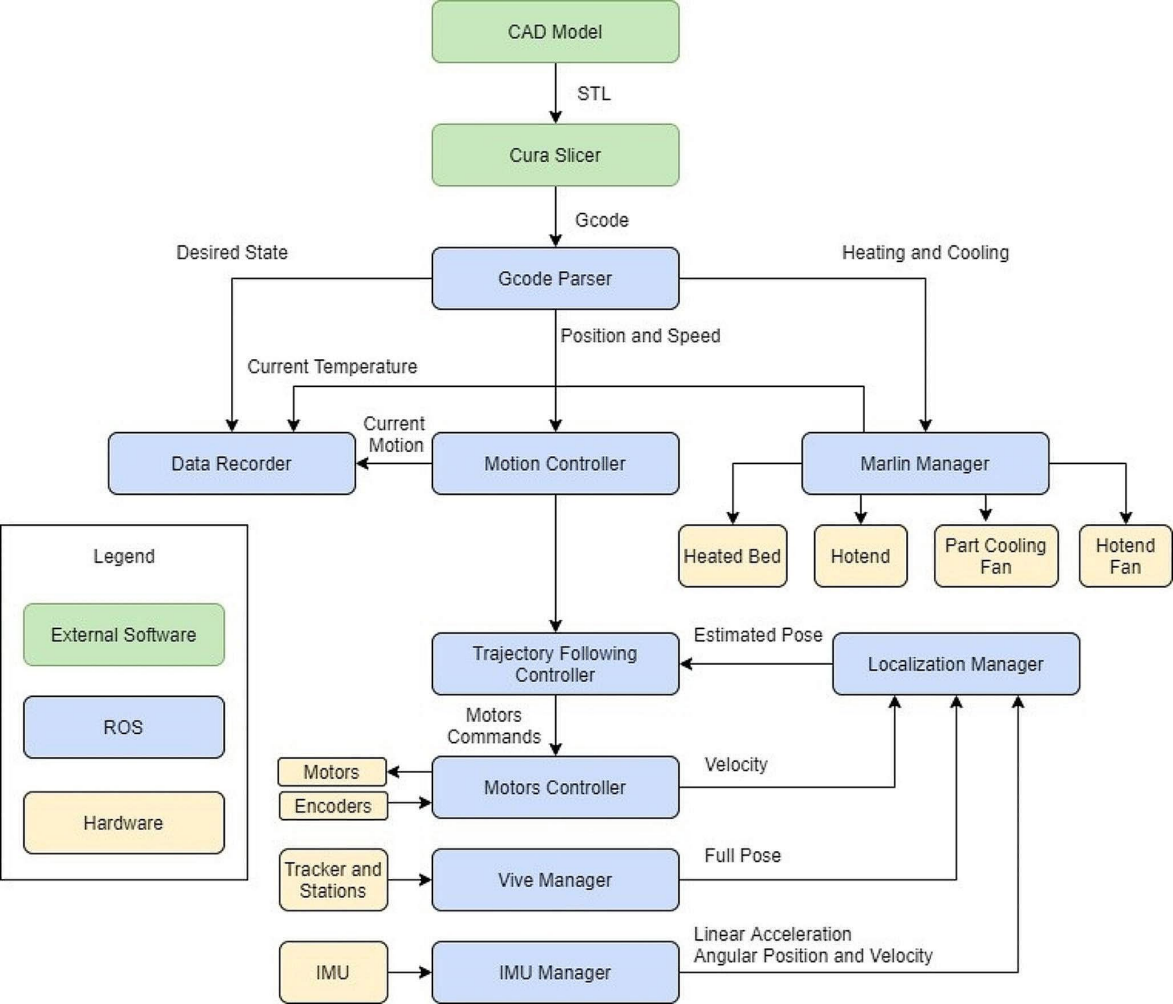
Information flow

The overall information flow, from a CAD model to the final 3D printing is shown in Fig. 5. In this case, the communication system for the mobile robot implementation is based on the Robot Operating System (ROS) and Marlin Firmware. ROS manages all aspects of the system except for the heating and cooling elements, which are controlled by the Marlin Firmware. Several scripts were developed on ROS:

- **Motion_Controller**: uses PID controller and trapezoidal trajectory generator to send the control commands to the “Motors_Controller” using the desired position from the “Gcode_Parser” and the current position from the “Localization_Manager”.
- **Motors_Controller**: directly communicates with each motor in the system, including the wheels’ motors, the Z-axis and the E-axis motors. It reads the current encoder values and writes the new velocities. It has the functionality to home the Z-axis using torque control, which stops the homing when the Z-axis touches the platform.
- **Localization_Manager**: subscribed to each sensor manager in the system to get its data, producing an estimated pose and velocity using the KF. Since the data comes at different rates, a custom message synchronizer was developed. It also calculates the required transformation for each sensor.
- **Vive_Manager**: implements the Libsurvive library to communicate with the Vive Tracker and the base stations. It attaches a function that is called whenever there is a new measurement from the system regarding the position or the velocity of the Vive Tracker. Since the Libsurvive library considers one of the base stations as the center of the world frame, it must be transformed in this script.
- **Gcode_Parser**: reads all the lines in a Gcode file and splits them into an array of lines that contains the G or M commands and other parameters such as temperature, position, and velocity. It also sends each line to its corresponding manager. For example, the bed heating M command (M190) is sent to the “Marlin_Manager” while a motion command (e.g. G1) is handled by the “Trajectory_Following_Controller”. When the marlin manager and the wheels controller send a signal informing that they are finished, this script goes to the next line, repeating the procedure until all lines are finished.
- **Marlin_Manager**: connects to the MKS Gen 1.4 electronic board that has the Marlin Firmware. It reads and writes values from and to the board and deals with the heating and cooling components.
- **Data_Recorder**: reads the data from all sensors and the controller and saves them as “.csv” for analysis purposes after finishing the printing process.

## Experimental results and discussion

This section investigates the accuracy of the proposed mobile robot and compares the 3D printing performance between the proposed system and a commercial gantry system. The 3D printing performance is assessed considering the quality of printed parts, dimensional accuracy, surface roughness, layer height, and mechanical properties. Moreover, potential areas to further optimize the proposed system are presented and discussed.

### Evaluation of sensor configurations and motion accuracy

#### Global sensor configurations

This section investigates the global sensor configurations in terms the noise accumulation during the sensor operation. The noise accumulation is a significant factor to consider given that AM is a process that operates over a long period of time in particularly when dealing with large objects or low printing speeds.

In the proposed system, the HTC Vive interacts with two notable libraries, OpenVR and Libsurvive (Lohr, 2022). The OpenVR, an official open-source library, is oriented towards VR for game developers and other VR applications. However, its core, SteamVR, is closed-source, preventing any modification or alteration of its computations. The pose of the tracker is sampled at a frequency of 90 Hz. A considerable drawback of OpenVR is its restriction on using Vive Trackers independently of an HMD device, which increases the computation requirements without providing commensurate benefits to the system. The Libsurvive, in contrast, is an unofficial, fully open-source library that primarily focuses on the tracking aspect of the VR system. Developed by the community with the intent to reverse engineer the HTC Vive completely, it allows for modification of any part of its computations, including calibration processes, filters, and posers, among others. Its tracker samples are collected at 130 Hz, a higher frequency compared to OpenVR. While both libraries enable users to obtain the pose of the tracked object, Libsurvive offers an added advantage by allowing the customization of pose calculations and filters.

Table 2 summarizes the outcomes of a static analysis conducted to evaluate the performance of the two libraries in tracking the position of a stationary device. As SteamVR (the core of OpenVR) prohibits user modification, only a single configuration was tested. Contrary, Libsurvive permits full computational customization. Multiple configurations were evaluated for Libsurvive, such as the default settings, or disabling filtering of sensors data, each performed with and without an HMD. The standard deviations of the reported positions across the three axes (X, Y, and Z) and the total standard deviation used to facilitate the comparison are listed for each configuration in the Table 2.

For the OpenVR, the standard deviation values range from 0.073 mm (Z-axis) to 0.099 mm (X-axis), with a total standard deviation of 0.252 mm. These values suggest a moderate level of variability in the system’s ability to accurately determine the device’s position.

Comparatively, Libsurvive shows differing performances based on its configuration settings. The use of Libsurvive with an HMD and default settings yielded slightly better accuracy than OpenVR, as evidenced by the lower total standard deviation of 0.196 mm. However, when the filter is removed with an HMD in place, the standard deviation remains relatively low (0.233 mm), suggesting the system’s resilience against positional uncertainty in the absence of filter assistance. On the other hand, the performance of Libsurvive without an HMD presents the lowest total standard deviation (0.155 mm) in the default settings scenario. This indicates that this configuration as the best configuration for accurate positioning. Even in the case of deactivating the filter under no-HMD conditions, the total standard deviation (0.222 mm) remains lower than that of OpenVR.

Overall, results presented in Table 2 indicate that the performance of Libsurvive can surpass OpenVR, particularly when used without an HMD and with default settings. Consequently, the chosen configuration for the mobile robot entails the use of Libsurvive with default calculations and without connecting an HMD.

**Table 2:** Standard deviation of positional accuracy for OpenVR and Libsurvive under different configuration settings

Method	Standard Deviation (in mm)			
	X-axis	Y-axis	Z-axis	Total
OpenVR	0.099	0.08	0.073	**0.252**
Libsurvive (w/ HMD) Default Settings	0.08	0.081	0.034	**0.196**
Libsurvive (w/ HMD) No Filter	0.058	0.111	0.064	**0.233**
Libsurvive (w/o HMD) Default Settings	0.045	0.073	0.037	**0.155**
Libsurvive (w/o HMD) No Filter	0.057	0.106	0.058	**0.222**

#### Evaluation of the mobile robot accuracy

To evaluate the motion accuracy of the mobile robot, a test was performed in which the robot was set to move a distance of 400 mm at a velocity of 20 mm/s. To accurately determine the robot’s actual path, a drawing apparatus was attached to its end effector. The subsequent path traced was then retrieved and digitally processed to determine any deviations from the intended trajectory.

**Fig. 6:**
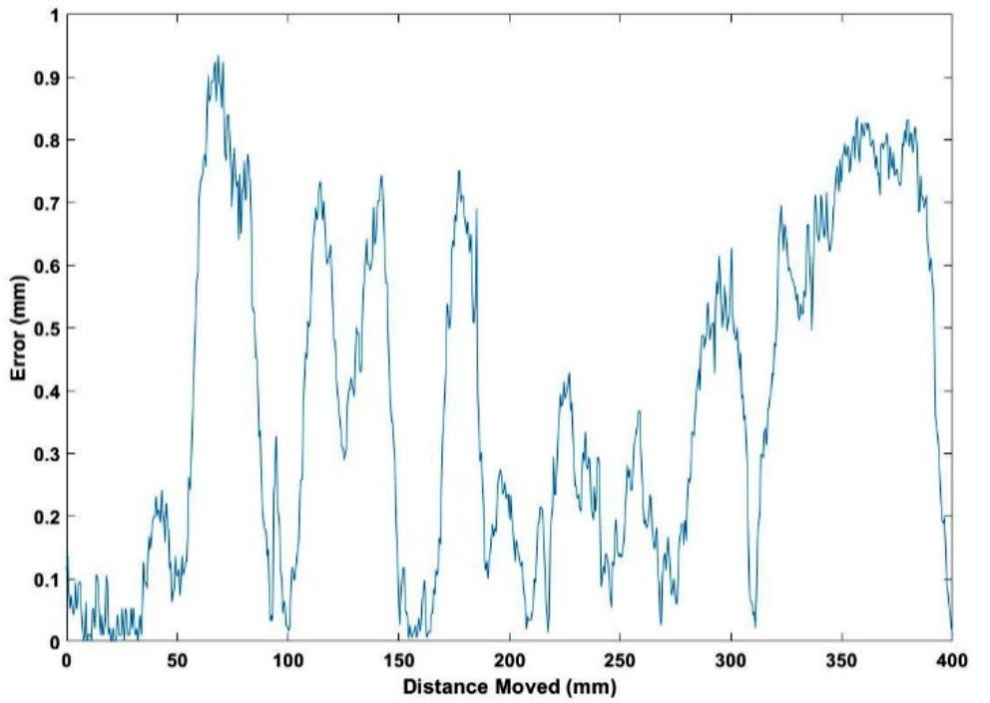
Absolute error versus total distance moved by the mobile robot

Figure 6 depicts the relationship between the absolute error and the total distance traversed by the mobile robot. During the initial 40 mm of movement, the robot demonstrated exceptional precision with minimal errors, averaging around 0.04 mm. However, subsequent motion started exhibiting bigger deviations from the intended path, with errors approximately reaching 0.93 mm upon completion of a total distance of 70 mm. Therefore, the accuracy displayed considerable fluctuation until the robot completed its designated movement. However, there is no visible accumulation of errors over time. As can be observed from Fig. 5, the average error rate remains fairly constant. Across the entire distance traversed, the average error value is approximately 0.37 mm.

The obtained results provide significant insights into the performance of the mobile robot for AM applications. The initial high precision achieved by the robot, as evidenced by the minimal errors during the first 40 mm of movement, shows its potential for performing highly accurate maneuvers. This level of accuracy is particularly crucial for AM where precise layer-by-layer deposition is key to part quality. However, the subsequent increment in error, peaking at about 0.93 mm at the 70 mm mark requires further improvements. This deviation from the intended path may be attributable to a variety of factors, such as mechanical drift or slippage. Despite these variations in precision, the error does not accumulate over time, as evidenced by the relatively constant average error rate. The maximum error observed, approximately half the size of the nozzle utilized, implies that even under conditions of maximum deviation, printing will not occur in mid-air, as roughly half of the current print line will contact the previously printed layer. Furthermore, the average error, corresponding to around 20% of the nozzle size, highlights the robot’s commendable accuracy. This suggests that even with longer tasks, the mobile robot’s error rate remains within an acceptable range, demonstrating its potential for successful deployment in material extrusion AM of plastic parts.

**Fig. 7:**
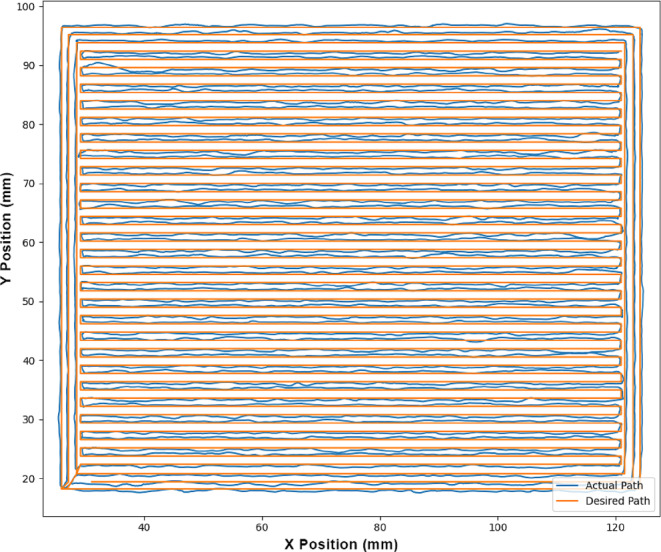
The desired and actual motion of the mobile robot while printing a rectangular layer

The mobile robot was assigned a 3D printing task, focusing on motion testing over larger distances, without actual material deposition. The task involved replicating the motions required to print a rectangular prism measuring 100 mm in length, 80 mm in width, and 10 mm in height. The operation spanned approximately one hour and covered a total distance of 39,013 mm. The task was completed successfully, with no accumulation of positional errors, underscoring the efficiency of the developed localization system in eliminating potential sources of error accumulation. Both the average and maximum errors observed align with the previously mentioned values. Fig. 7 illustrates the first layer of the object for clarity, with subsequent layers omitted to simplify the visual presentation.

Table 3 presents a comparative analysis of positional accuracy among other mobile 3D printers as reported in the literature, including the findings from our work. Notably, our work distinguishes itself by achieving submillimeter accuracy in both average and maximum error measurements, a milestone not reported by any other published works. While the literature reports average errors ranging from 1.23 mm to 2.20 mm, our work substantially lowers this to 0.37 mm, approximately a reduction of 70% compared to the next best reported average error in the table. Similarly, the maximum error in previous works spans from 3.54 mm to 9.90 mm, whereas our research achieves a maximum error of just 0.93 mm. This shows the capability of the developed mobile robot for accurate 3D printing.

**Table 3:** Average and maximum errors for different mobile 3D printers in the literature and our work

Mobile 3D Printer	Average Error	Maximum Error
(Xu et al., 2021)	1.67 mm	7.30 mm
(Tiryaki et al., 2020)	2.20 mm	9.90 mm
(Marques et al., 2017)	1.23 mm	3.54 mm
Our work	0.37 mm	0.93 mm

### 3D printing with the mobile robot

In this section, 3D printing results using the developed mobile robot are presented and discussed. Two cases (rectangular prisms and cylindrical parts) were considered to investigate key parameters such as accuracy, surface roughness, overall shape fidelity, and mechanical properties. Key advantages and limitations of the proposed system are also discussed. For all considered cases, parts were printed using both the developed mobile 3D printer and a gantry 3D printer. The gantry 3D printer considered in this study was the commercial desktop 3D printer Original Prusa i3 MK3S+ (Prusa Research, Czech Republic). This 3D printer was modified to have similar depositing hardware (same nozzle, hotend, and hotbed as the mobile 3D printer). This modification allowed both machines to produce similar parts in ideal scenarios. Table 4 presents the main print settings, including the motion, temperature, and material settings.

In assessing the quality of the 3D printed parts, we adopt a multi-faceted approach to ensure a comprehensive evaluation:

- Dimensional accuracy, gauged against CAD models, reflecting the accuracy of the prints. The samples were measured ten times, and the mean and variations were taken from those measurements. As previously stated by Ravi et al. (2022), a good dimensional accuracy should be within 1% of the highest length of the part, or 1 mm in the case of the prints presented in this work.
- Surface roughness, a critical aspect of finish and aesthetic appeal, is quantified using a scanning electron microscope. A good quality part should have ${R}_{a}$ < 3 μm (Lalegani Dezaki et al., 2021).
- Layer adhesion and consistency are also visually and quantitatively analyzed for structural integrity.
- Geometrical fidelity is also considered, ensuring the printed parts replicate the designs without warping or deformation.
- A visual inspection complements these measures, offering a holistic view of quality and revealing subtle defects not captured by numerical metrics.

**Table 4:** Material and print settings for both mobile and gantry 3D printer

Setting	Value
Material	PLA (White)
Layer height	1 mm
Line width	1.4 mm
Wall count	2
Top and bottom pattern	0 and 90 Lines
Infill pattern	Lines
Infill density	15%
Hotend temperature	225 °C
Heated bed temperature	55 °C
Speed	20 mm/s

#### Part quality and dimensional accuracy

**Fig. 8:**
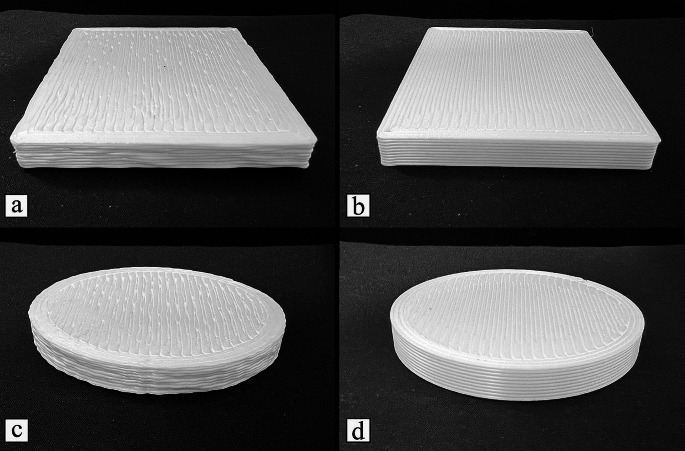
Rectangular prism printed by (**a**) mobile 3D printer and (**b**) gantry 3D printer. And Cylindrical parts printed by (**c**) mobile 3D printer and (**d**) gantry 3D printer

The first testing part is a rectangular prism with dimensions 100 mm x 80 mm x 10 mm (length x width x height). The second testing part printed is a cylinder with 80 mm of diameter and 10 mm of height. Results are presented in Fig. 8 for both parts, while Table 5 compares the designed and the obtained dimensional values.

**Table 5:** Dimensions of the parts produced by both mobile and gantry 3D printers. Units in mm

	Dimension	CAD Model	Gantry 3D Printer	Mobile 3D Printer
Rectangular Prism	Length	100	99.68 ± 0.13	100.48 ± 0.46
	Width	80	79.73 ± 0.11	80.46 ± 0.26
	Height	10	9.69 ± 0.10	9.70 ± 0.11
Cylindrical Part	Diameter	80	79.83 ± 0.15	80.32 ± 0.39
	Height	10	9.81 ± 0.07	9.77 ± 0.13

In comparison to the results reported for other plastic mobile 3D printers (Marques et al., 2017; Milkert, 2014; Xu et al., 2021), the proposed mobile 3D printer was able to fully print the parts without any issues, such as drifts, incomplete print, or poor quality parts. Knowing that each of those parts took at least 1 h to print, this is an indicator that the mobile robot is reliable as a 3D printer. Moreover, results show that the developed system is well calibrated as there was no drift over the printing duration. Besides, the overall shape of both the rectangular prism and the cylindrical parts produced by the mobile robot match the CAD model. Furthermore, the dimensional accuracies of both parts are comparable to the parts produced by the commercial gantry system.

The rectangular prism printed by the mobile robot (Fig. 8a) presented some errors shared by the part printed by the commercial gantry 3D printer (Fig. 8b). First, the corners are not sharp, presenting a small fillet radius, which can be attributed to the large nozzle size used and the printer spending more time near the corners. However, this is more visible in the part printed by the mobile robot. Additionally, the rectangular prisms printed by both the mobile and gantry systems have a larger first layer. This issue is known as Elephant Foot (Lowke et al., 2020), which results from the weight of the part compressing the first layer. However, this issue can be reduced by increasing the cooling and decreasing the hotbed temperature. Moreover, the top layer of the part printed by the mobile robot shows a net effect due to some gaps between the printed lines. However, this can be solved by reducing the line width, so extrusion lines are near to each other or overlap during noisy motion.

The rectangular prism produced by the mobile 3D printer exhibits other errors not visible on the part printed by the gantry system. Overall, the rectangular prism model presents slightly worse quality and shape fidelity compared to the gantry part. First, the extrusion lines are jagged, which are noticeable by observing the rectangular prism from the top or bottom. Furthermore, the parts produced by the mobile robot exhibit irregular layer heights when observed from the sides, which can be attributed to the way the mecanum wheels work.

As expected, results (Table 5) showed that the gantry 3D printer had the lowest variations. The mobile robot showed a slightly higher range. The mean of the length and width of the part of the gantry 3D printer is 0.32 mm and 0.27 mm lower than the optimal, respectively. On the other hand, the rectangular prisms produced by the mobile robot are averaging around 0.47 mm increase over the optimal size. The heights of all the rectangular prisms are almost similar.

The cylindrical parts produced by both machines (Fig. 8c and d) showed similar results to the ones observed for the rectangular prisms. One defect shown in the backside view of the cylindrical parts printed by both systems is a hollow column near the start of the printing path, which is the Z seam effect. Moreover, the lines of the cylindrical part fabricated by the mobile robot are more jagged than those of the prism. This is due to the many short lines that are generated by the STL file to create curved lines, stopping the mobile robot momentarily between the lines. As observed from Table 5, the diameter of the part printed by the mobile robot is closer to the CAD model than the length and width of the rectangular prism. However, the diameter variations are still large for the part produced by the mobile robot compared to the variation of the part produced by the gantry 3D printer.

**Fig. 9:**
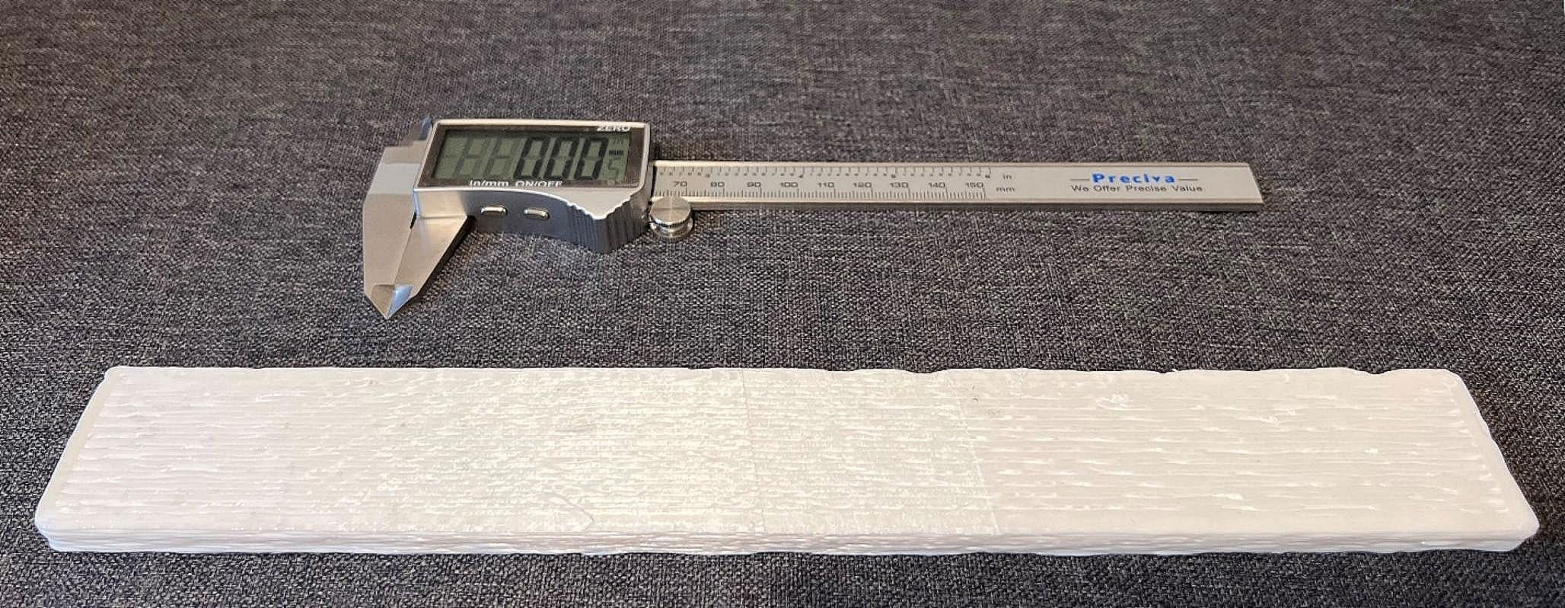
A printed object larger than the mobile 3D printer

The final conducted test aimed to investigate the ability of the mobile robot to print large-scale objects. In this case, the mobile robot was tasked with printing a rectangular object with a 300 mm length (Fig. 9), which is around 36% larger than the length of the robot itself. In this case, a different heated bed was used as the size of the bed is smaller than the length of the printed object. This shows the mobility advantage of mobile 3D printing, as the machine size of the gantry 3D printer used in this study is 500 mm in length with a printing area designed to be square with 210 mm side length, thus the workspace-to-machine length ratio is 0.42 which is 69% less than the developed mobile 3D printer.

#### Surface roughness and layer height

The rectangular prisms printed by both the mobile and the gantry systems were further investigated in terms of the layer heights and the surface roughness (top surface). Samples were sectioned using a Buehler Isomet 5000 (Buehler, US), with an abrasive silicon carbide blade, which ground the material away while ensuring smoother cuts. Samples were subsequently sputter-coated with gold using an Emscope SC 500 and analysed using the scanning electron microscope (SEM) TM3000 Tabletop Hitachi (Hitachi, Japan) at low magnification (x40), to measure differences in layer heights. Obtained images were analysed using the Fiji software. Surface roughness was determined using an Alicona G4 Infinite Focus (IF) microscope (Bruker Alicona, Austria). The magnification was set to x5 for all samples to keep consistency and to investigate differences in surface integrity between gantry and mobile robot printed samples.

**Fig. 10:**
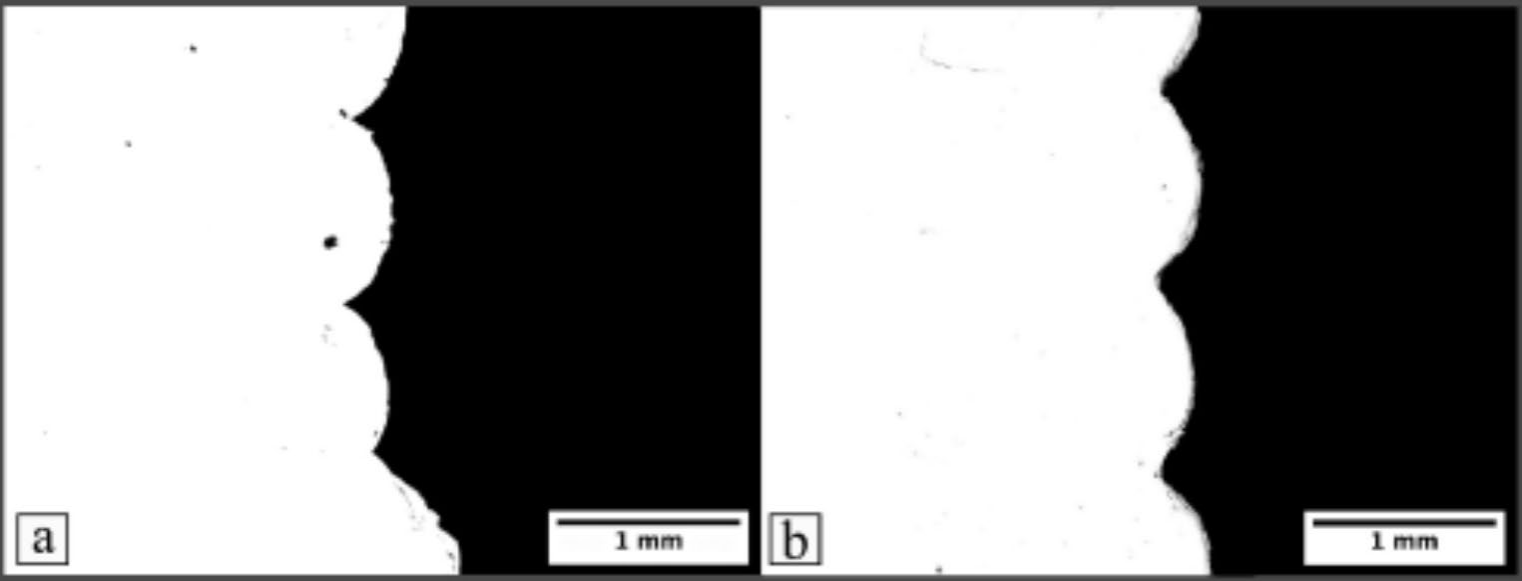
SEM images showing the side view of the layers of the parts printed by (**a**) the mobile 3D printer, and (**b**) the gantry 3D printer

Figure 10 shows the layering patterns of parts printed by both mobile and gantry systems, revealing two complete layers and two partial layers, resulting in three discernable layer heights. The mean layer heights for samples printed by the mobile robot and the gantry system were found to be 1.016 mm and 1.009 mm, respectively, supporting the aforementioned results that indicate the mobile robot achieves comparable layer heights to the conventional gantry system. The variations in layer width were 0.1340 mm and 0.0252 mm for the rectangular prisms printed by the mobile robot and the gantry system, respectively. Upon excluding the lower layer of the mobile robot part, the average deviation narrows to 0.0297 mm, closely resembling the value observed for the parts printed by the gantry system. The primary cause for the variation in layer widths observed in the parts printed by the mobile robot can be attributed to motion noise in the XY plane. Moreover, it is noteworthy that the layers printed by the mobile robot appear more compressed than those produced by the gantry system, which may be a consequence of vertical vibrations caused by the mecanum wheel.

**Fig. 11:**
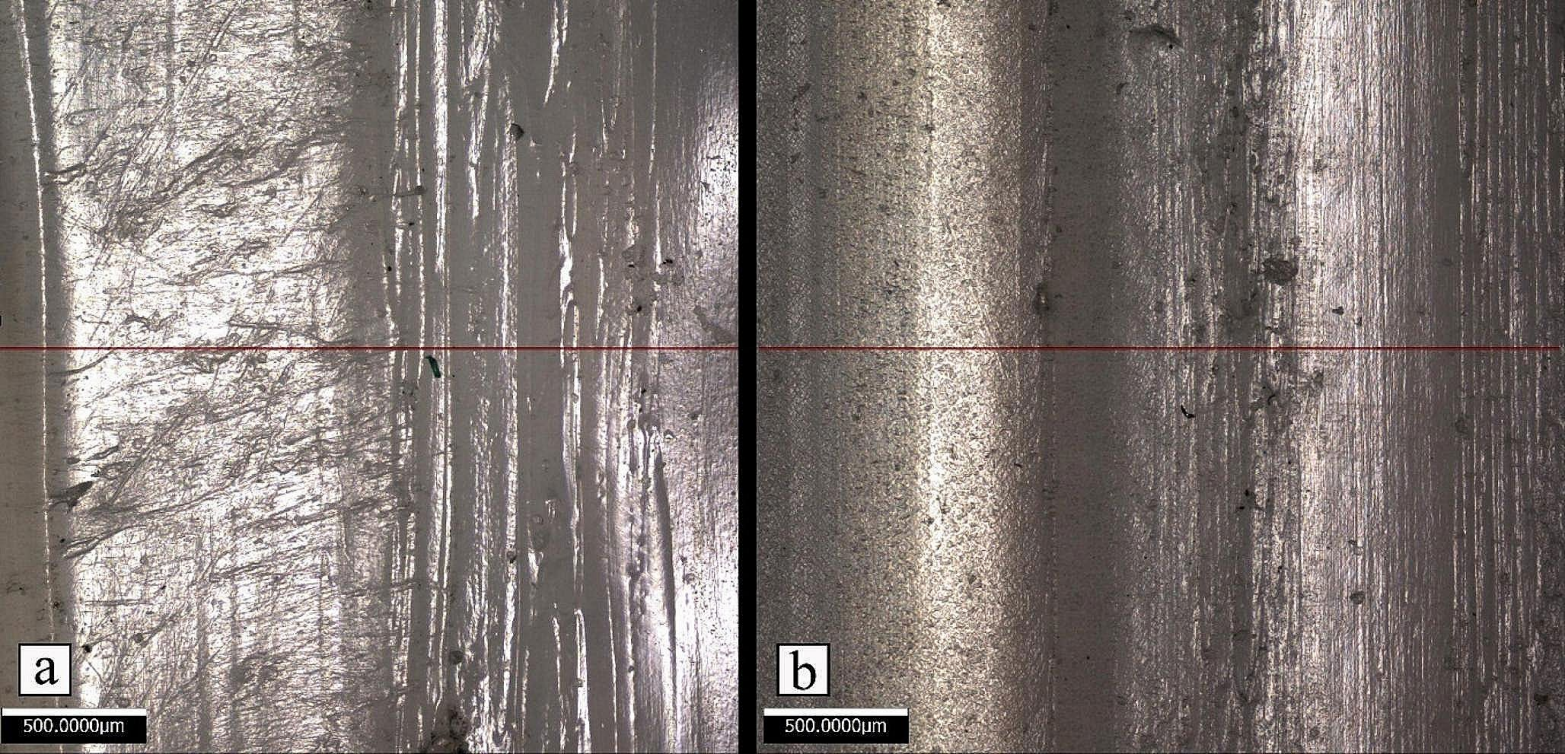
Surface and profile of the rectangular prisms printed by (**a**) the mobile 3D printer; (**b**) the gantry 3D printer

Figure 11 presents the top surfaces of samples printed by both systems, highlighting the profile lines used for surface roughness measurements. As observed, parts produced by the gantry system exhibit greater uniformity in comparison to the mobile robot’s counterparts. Nevertheless, the average roughness of the profile (${R}_{a}$) for the parts printed by the mobile robot is 2.238 μm while the parts printed by the gantry system showed a value of 1.447 μm. Other values for the roughness of parts printed by both systems are presented in Table 6, which shows that the mobile robot printed part has higher roughness values.

**Fig. 12:**
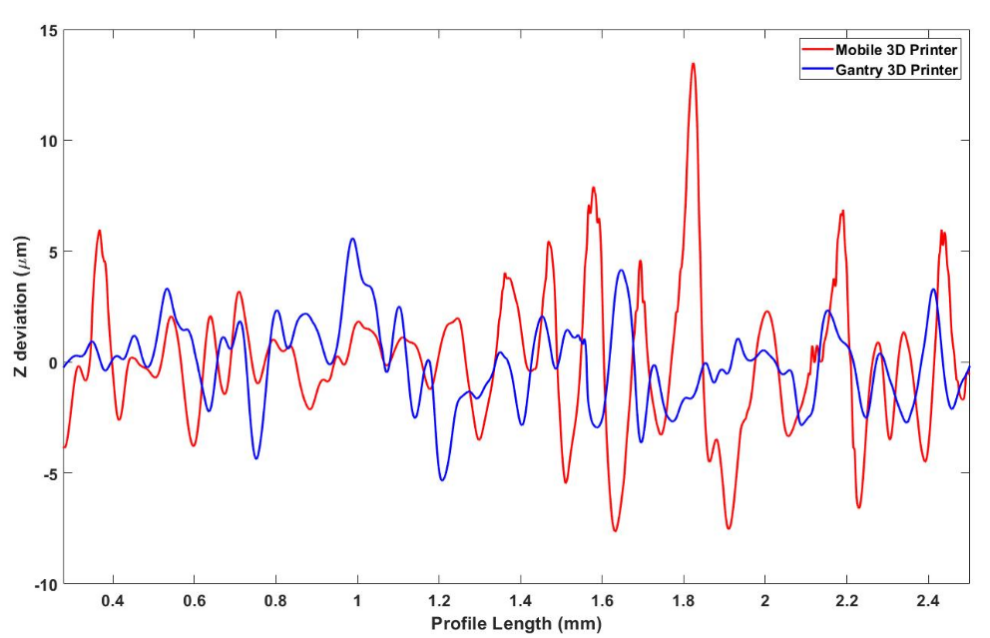
Z deviation of the surface and profile shown in Fig. 11

Figure 12 shows the height deviations across the length of the profiles for both parts. The relationship between layer thickness and surface roughness, as observed by Wu et al. (2017) and Ayrilmis (2018), also confirms that an increase in layer thickness leads to greater surface roughness and a more pronounced stair-like appearance on the external profile. The increase in layer thickness also negatively affects the accuracy and precision of the 3D printed part’s final shape. In general, parts fabricated by the mobile robot exhibit slightly worse results than the gantry-produced parts. However, numerous pronounced peaks are particularly evident on the right side of the profile, which can be attributed to the random vibrations generated by the wheels.

**Table 6:** Surface Roughness values for both mobile and gantry 3D printer. ${R}_{a}$ is the average roughness of the profile, ${R}_{q}$ is the root-mean-square roughness of the profile while ${R}_{z}$is the mean peak to valley height of the profile

Printing System	Surface Roughness Values		
	Ra (µm)	Rq (µm)	Rz (µm)
Gantry Printer	1.447	1.866	7.371
Mobile Robot Printer	2.238	3.102	12.439

**Fig. 13:**
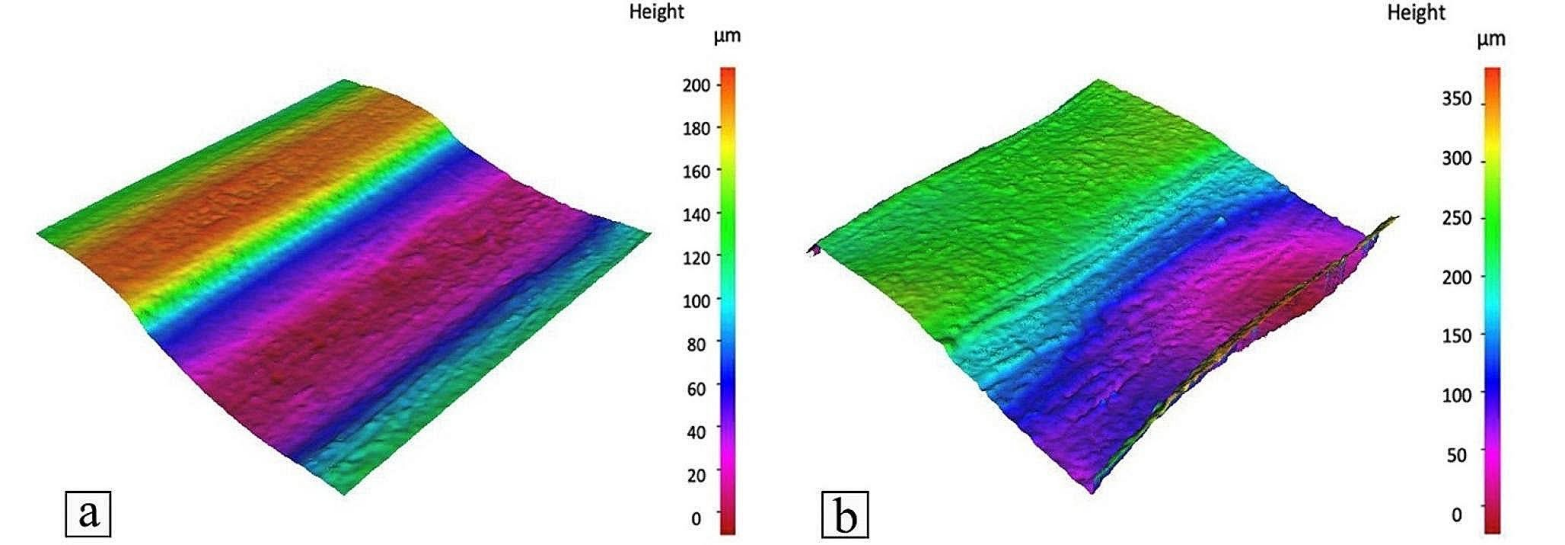
The 3D Surface roughness maps for parts printed by (**a**) gantry 3D printer, and (**b**) mobile 3D printer

Figure 13 also shows the 3D surface roughness maps for the parts printed using both systems. The surface roughness of the parts printed using the gantry 3D printer, as illustrated in Fig. 13a, display a relatively consistent pattern with smoother transitions between layers. The ridges and valleys are well-defined but do not exhibit significant abrupt peaks or deep troughs. This is consistent with the reported ${R}_{a}$ of 1.447 μm, which suggests a finer surface finish where the deviations from the mean line are less pronounced.

In contrast, Fig. 13b, which represents the output from the mobile 3D printer, shows a rougher texture with greater height variability. The range of colors indicates a more significant variation in height across the surface, correlating to the higher ${R}_{a}$ value of 2.238 μm, previously reported. This indicates a surface with more pronounced deviations from the mean line, reflecting a rougher texture that might be attributed to the dynamic nature of the mobile 3D printer’s operation, including factors such as movement precision, and layer adhesion variability.

#### Mechanical properties

**Fig. 14:**
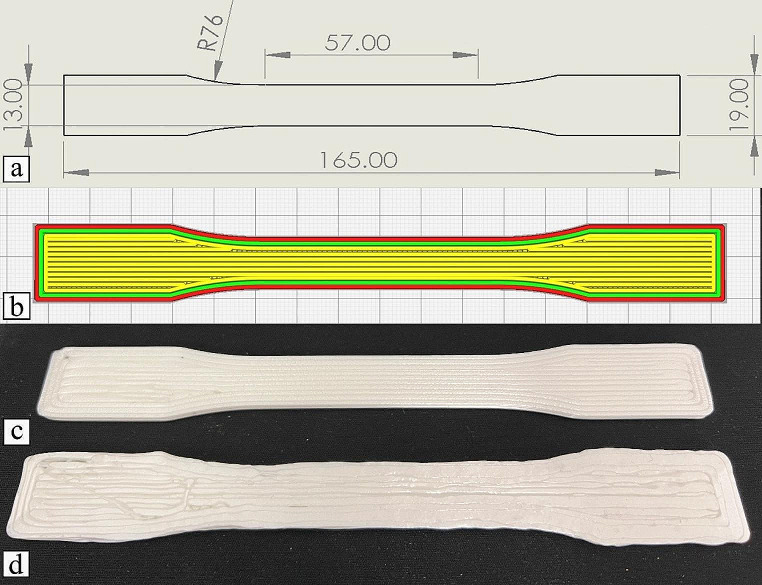
(**a**) Dimensions of the specimens (**b**) slicer view of the toolpaths, (**c**) a specimen printed by the gantry printer, and (**d**) a specimen printed by the mobile printer

The mechanical properties of parts produced by the developed mobile robot and the gantry system were investigated by printing test specimens according to standard Type 1 of ASTM D638 (ASTM, 2015). Figure 14 shows the dimensions of the specimens, the slicer view of the toolpaths, and two specimens printed by both printing systems.

All samples were printed using the same process parameters. Parts were printed in PLA with ± 0.02 mm precision from Prusament (Prusa Research, Czech Republic). The printing temperature and the platform temperature were 225 °C and 55 °C, respectively, and the printing and travel speed were 20 mm/s. The printing line width was changed to 1.2 mm to include as many lines as possible in the middle section. Moreover, as the thickness of the specimen is 3 mm, the layer height was chosen to be 1 mm. The STL file was sliced such that the bottom and the top layers were oriented with the longitudinal axis of the specimen. The middle layer was sliced with 100% infill but oriented perpendicular to the other layers.

Mechanical tests were conducted using the Instron 5967 universal testing machine (Instron, US), equipped with MTS 634.31 F-24 axial extensometer to measure the elongation of the specimens. The speed of testing dictated by the standard was 5 mm/min. Seven specimens were printed per printing system.

**Fig. 15:**
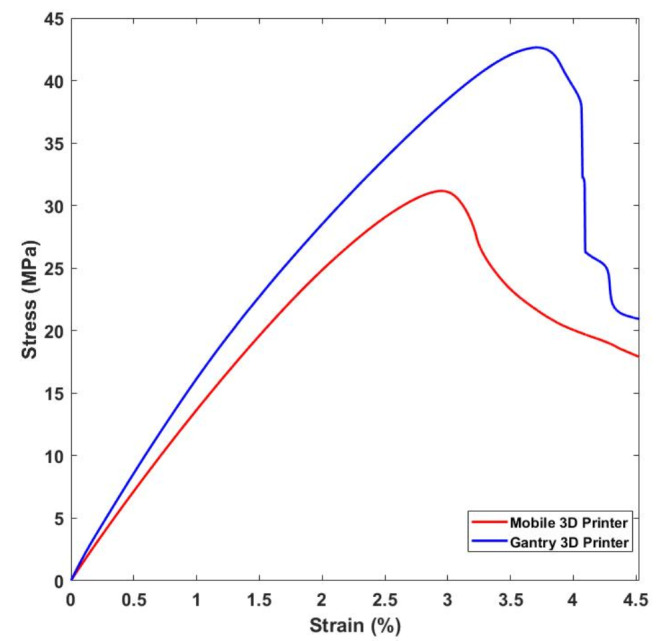
Stress-strain curves for specimens produced by the gantry and mobile 3D printers

Figure 15 shows the average of the tensile test results and Table 7 shows the values. As observed the specimens printed by the mobile robot show lower elasticity compared to those printed by the gantry system. The maximum tensile strength for the parts printed by the gantry 3D printer was 42.57 ± 1.93 MPa, while for the mobile 3D printer the maximum tensile strength was 31.16 ± 2.31 MPa. This can be attributed to the vibrated motion of the mobile robot that creates more regions with small voids which result in a lower contact surface between the layers. Voids are also generated due to the fluctuations of the accuracy. It has been reported that voids in 3D printed parts affect the mechanical properties significantly (Rimašauskas et al., 2022). The specimens printed by the mobile system presented in average 11% less elongation. The elastic modulus of the parts produced by the gantry and mobile robots were 1942 ± 47.86 MPa and 1501 ± 78.53 MPa, respectively.

**Table 7:** Mechanical properties values for both mobile and gantry 3D printer

Property	Gantry 3D Printer	Mobile 3D Printer
Tensile Strength	42.57 ± 1.93 MPa	31.16 ± 2.31 MPa
Elastic Modulus	1942 ± 47.86 MPa	1501 ± 78.53 MPa

#### Comparison to other works in literature

**Fig. 16:**
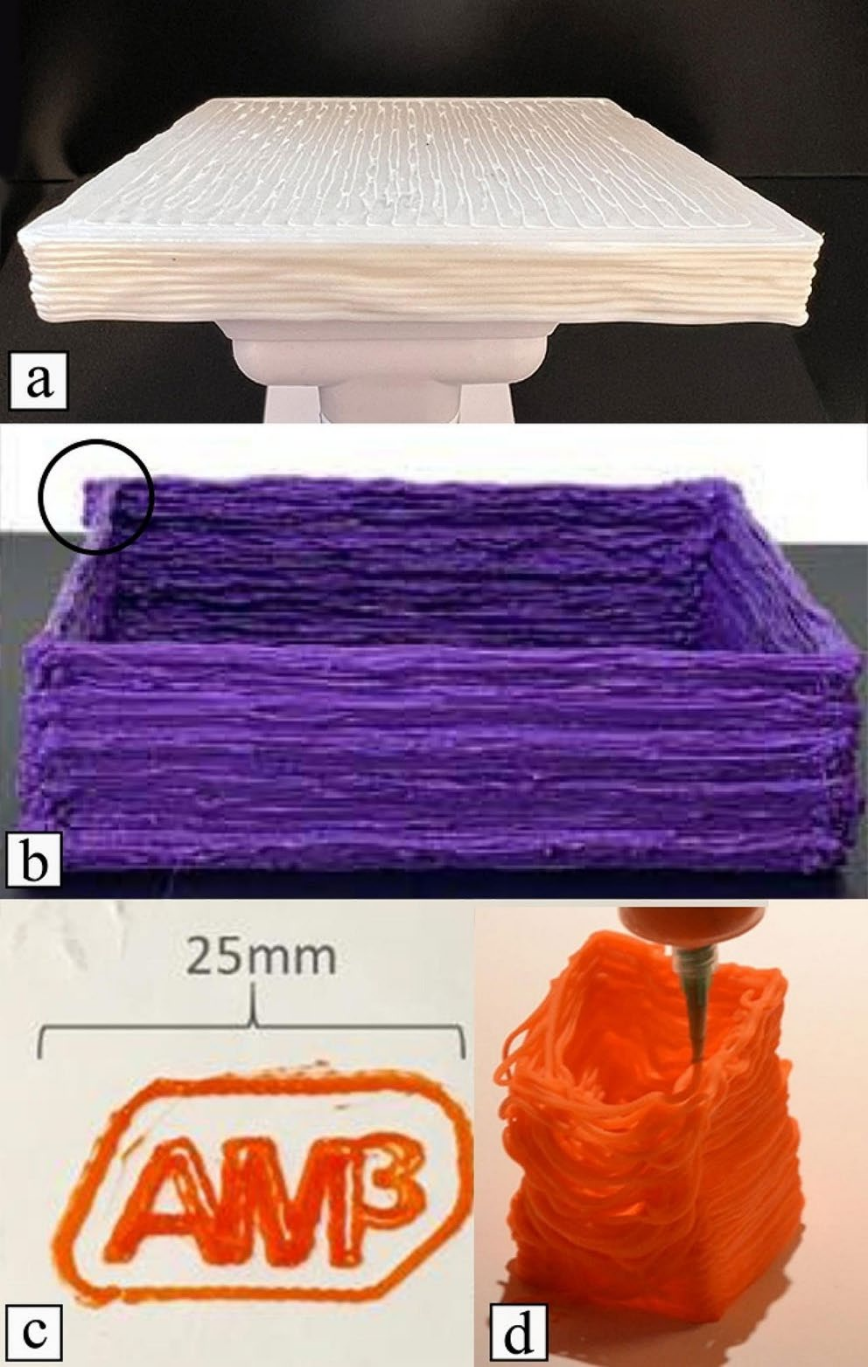
Current attempts at plastic 3D printing with mobile robots. (**a**) Our work, (**b**) Projector-Guided Mobile Robot (Xu et al., 2021), **c**) AM^3^ (Marques et al., 2017), and (**d**) 3&Dbot (Milkert, 2014)

The performance of the proposed mobile 3D printing system and other print-while-moving systems for plastic printing was also investigated. Three cases (Fig. 16) were considered.

As observed, our work (Fig. 16a) is the only one that printed a fully enclosed 3D object, while the other reported works either print an extruded perimeter (Fig. 16b and d) or few layers (Fig. 16c). In the Projector-Guided Mobile Robot (PGMR) system (Fig. 16b), a rough surface is noticeable in the sides (Xu et al., 2021). This is likely due to noisy motion when moving in a straight line, which is not so significant in the parts printed by the proposed mobile system. Another issue observed in the PGMR system is when two lines touch, which creates a blob of plastic at the intersection point. This issue is clear at the corners, particularly at the back corners of the printed part, as indicated within the circled area in Fig. 16b. In the case of the AM3 mobile robot (Marques et al., 2017), authors only printed three layers (Fig. 16c). However, their mobile robot has a Z-axis that makes use of linear shafts of 200 mm in length. Using a large Z-axis length and only printing three layers seems to indicate that the system is not capable of printing 3D parts with a large number of layers. A similar issue can be observed in the work reported by Duan et al. (2019). Moreover, in the printed 3-layer part, the extrusion lines are not fully filling the area inside the word “AM3”, and the lines exhibit some irregularities, with several visible gaps within the layer. Finally, the results reported for the mobile robot 3&Dbot system also show several limitations (Fig. 16d) (Milkert, 2014). Firstly, the layers are not sticking to each other. Secondly, the motion of the robot has a slight drift over time, which is clear when inspecting the top layers.

This research also investigates the dimensional accuracy of the printed parts. While the other works do not provide comprehensive dimensional details, the PGMR work shown in Fig. 16b reported a part thickness significantly exceeding the CAD model’s specified 2 mm, with an average error of 1.02 mm and a maximum deviation of 2.17 mm. In comparison, our work showcased an average length deviation of 0.48 mm with a maximum deviation of 0.94 mm, as detailed in Table 5, which shows that the proposed system presents better performance in maintaining dimensional accuracy.

Based on these results, it seems evident that the proposed mobile robot 3D printer surpasses all the current attempts of plastic 3D printing by other mobile robots and is the closest to gantry 3D printers.

## Conclusions

This study introduces a novel mobile 3D printer for material extrusion AM and represents an advancement in the domain of accurate mobile 3D printing. The research undertaken not only challenges the traditional constraints associated with mobile 3D printers but also opens new possibilities for on-site and large-scale manufacturing. The key scientific outcomes of this research are:

- The motion accuracy evaluation revealed an average error of approximately 0.37 mm across the entire distance traversed for the mobile robot, indicating a reliable and consistent performance that is crucial for the layer-by-layer deposition process of AM.
- The quality and dimensional accuracy of printed parts by the mobile 3D printer were rigorously compared to those printed by a commercial gantry system. Results indicated that the mobile 3D printer can produce parts with slightly similar quality to those printed by a gantry system and significantly better than other plastic mobile 3D printers reported in the literature. Furthermore, the dimensional accuracy exhibited deviations less than 0.5 mm from the designed model, demonstrating a high level of accuracy in the printing process.
- The capability of the mobile 3D printer to print objects larger than itself (36% larger than its size) was successfully demonstrated. This paves the way for onsite and large-scale manufacturing and construction applications.
- The comparative analysis of surface roughness and mechanical properties showed that the mobile 3D printer, while below the gantry printer, still achieves satisfactory quality. More specifically, the mobile printer exhibited an average surface roughness of 2.238 μm, compared to 1.447 μm for the gantry printer. Moreover, the mobile printer’s parts reached approximately 73% of the gantry printer’s tensile strength.

Building upon the outcomes of this work, future work aims to refine and expand the capabilities of mobile 3D printing technology addressing the vibrational issues associated with mecanum wheels, which have been identified as the primary source of inaccuracies in the Z-axis and surface roughness. Solutions may include advanced vibration damping techniques or alternative wheel designs. Besides, the mobile 3D printer’s reduced tensile strength, compared to the gantry system, points towards a potential area for improvement. Future work, in particular for large-scale printing, should investigate the correlation between material properties and printing parameters to enhance the inter-layer adhesion and overall strength of printed parts, by optimizing the complex cooling-remelting-cooling sequence (crystallization and recrystallisation phenomena) associated to the layer-by-layer process.
